# Supplementary material for: Incorporating temporal distribution of population-level viral load enables real-time estimation of COVID-19 transmission
Source: Nat Commun. 2022 Mar 3;13:1155. doi: 10.1038/s41467-022-28812-9 (PMC8894407; doi:10.1038/s41467-022-28812-9)
Supplement: Supplementary file 1 — Supplementary Information [file 41467_2022_28812_MOESM1_ESM.pdf]

Supplementary information

Incorporating temporal distribution of population-level viral load enables real-time estimation of COVID-19 transmission

Yun Lin*, Bingyi Yang*, Sarah Cobey, Eric H. Y. Lau, Dillon C. Adam, Jessica Y. Wong, Helen S. Bond, Justin K. Cheung, Faith Ho, Huizhi Gao, Sheikh Taslim Ali, Nancy H. L. Leung, Tim K. Tsang, Peng Wu, Gabriel M. Leung, Benjamin J. Cowling

*Equal contribution

Correspondence to:
Benjamin J. Cowling (bcowling@hku.hk)

Supplementary Tables

Supplementary Table 1. Spearman's correlation coefficients (ρ) between Ct and the natural log-transformed incidence-based R_t over the third and fourth waves of COVID-19 in Hong Kong.

	**Wave 3 (Jul - Aug 2020)**		**Wave 4 (Nov 2020 – Mar 2021)**	
	ρ	***P*-value^**	ρ	***P*-value^**
**Ct mean**	-0.79	<0.001	-0.52	<0.001
**Ct skewness**	0.80	<0.001	0.27	0.002

^ Two-sided P-values that were rounded to 3 decimal places.

Supplementary Table 2. Associations between population Ct distributions and incidence-based R_t. Regression coefficients β, their 95% confidence intervals (CIs) and P-values were computed from the log-linear regression model shown in Eq. (7).

	β (95% CIs)	P-value^	Adjusted R square*
**Main model (training period between 6 Jul to 31 Aug 2020)**			
**Ct mean**	0.86 (0.81,0.92)	<0.001	0.72
**Ct skewness**	1.75 (1.11,2.75)	0.016	
**Validation model (training period between 20 Nov to 19 Dec 2020)**			
**Ct mean**	0.89 (0.83,0.95)	0.002	0.71
**Ct skewness**	1.57 (1.1,2.24)	0.014	

*Adjusted R square of the corresponding model.
^Two-sided P-values that were derived from t-tests for whether the coefficient was significantly different from zero, and were rounded to 3 decimal places.

Supplementary Table 3. Comparing Akaike information criterion (AIC) of regression models using different measurements for population-level Ct distributions.

Covariates included	Log-linear model*	Linear model*
Daily mean of Ct	36.93	56.35
Daily median of Ct	35.52	60.96
Daily skewness of Ct	52.25	84.97
Daily mean and skewness of Ct	32.75	57.77
Daily median and skewness of Ct	35.44	62.96

*For linear models, we used incidence-based R_t as the dependent variable; for log-linear models, we used natural log-transformed incidence-based R_t as the dependent variable.

Supplementary Table 4. Consistency between incidence-based and Ct-based method (as measured by Spearman correlation ρ) under various daily sample counts.

	All			Training period			Testing period		
**Sample count**	**Samples**	$\boldsymbol{\rho}$	***P*-value^**	**Samples**	$\boldsymbol{\rho}$	***P*-value^**	**Samples**	$\boldsymbol{\rho}$	***P*-value^**
(0,15]	67/213(31%)	0.40	0.002	11/62(18%)	0.37	0.497	56/151(37%)	0.37	0.009
[16,30]	36/213(17%)	0.48	0.004	12/62(19%)	0.52	0.089	24/151(16%)	0.35	0.092
[31,59]	42/213(20%)	0.76	<0.001	13/62(21%)	0.76	0.004	29/151(19%)	0.53	0.003
[60,130)	68/213(32%)	0.91	<0.001	26/62(42%)	0.82	<0.001	42/151(28%)	0.93	<0.001

^ Two-sided *P*-values that were rounded to 3 decimal places.

Supplementary Table 5. Associations between population Ct distributions and/or onset-to-sampling delay, and incidence-based R_t. Regression coefficients β and their 95% CIs were computed from log-linear regression models.

**Training period**	**Main (6 Jul to 31 Aug 2020)**		**Alternative (20 Nov to 19 Dec)**	
	**β (95% CIs)**	**Adjusted R square**	**β (95% CIs)**	**Adjusted R square**
**Ct alone (main model#)**		0.72		0.71
Ct mean	0.86 (0.81, 0.92)		0.89 (0.83, 0.95)	
Ct skewness	1.75 (1.11, 2.75)		1.57 (1.10, 2.24)	
**Ct and delay**		0.72		0.73
Ct mean	0.87 (0.82, 0.93)		0.92 (0.85, 0.99)	
Ct skewness	1.65 (0.99, 2.75)		1.52 (1.07, 2.17)	
Delay mean	0.93 (0.87, 1.01)		0.93 (0.80, 1.08)	
Delay skewness	1.00 (0.84, 1.18)		1.11 (0.97, 1.27)	
**Onset-to-sampling delay alone**		0.26		0.54
Delay mean	0.79 (0.71, 0.88)		0.72 (0.64, 0.81)	
Delay skewness	1.08 (0.84, 1.38)		1.16 (0.98, 1.37)	

#Main model used as in Eq. (7).

Supplementary Table 6. Parameters for the SEIR model.

Names	Description	Values
R_0	Basic reproductive number	2.2, 0.3, 1.9 and 0.3
t_0	Effective seeding time	2020-07-01
N	Initial population size	7,500,000
I_0	Proportion of individuals infected at seeding time	0.001%
$1/\sigma$	average time for individuals to transit from exposed-but-not-yet-infectious (E) to infectious (I)	5 days
$1/\gamma$	Average time for individuals to transit from infectious (I) to loss of infectiousness (mean infectious period)	4 days

Supplementary Table 7. Consistency between Ct-based R_t and simulation truth evaluated by the Spearman correlation ρ under each scenario for 100 runs.

**Scenarios#**	**Spearman ρ (95% CI)**		
	**Training period**	**Testing period**	**Testing period, over days > 30 daily records**
1 (flat detection at 25%)	0.9(0.86,0.93)	0.73(0.66,0.78)	0.74(0.67,0.79)
2 (flat detection at 10%)	0.84(0.74,0.92)	0.6(0.52,0.68)	0.75(0.65,0.84)
3 (detection increasing from 15% to 60% in the 2nd wave)	0.89(0.82,0.94)	0.77(0.73,0.81)	0.77(0.73,0.81)
4 (certain degree of under detection in the 2nd wave)	0.88(0.8,0.94)	0.65(0.53,0.75)	0.71(0.62,0.79)

#Detailed descriptions of each scenario were shown in Methods and in Supplementary Fig. 9.

Supplementary Figures

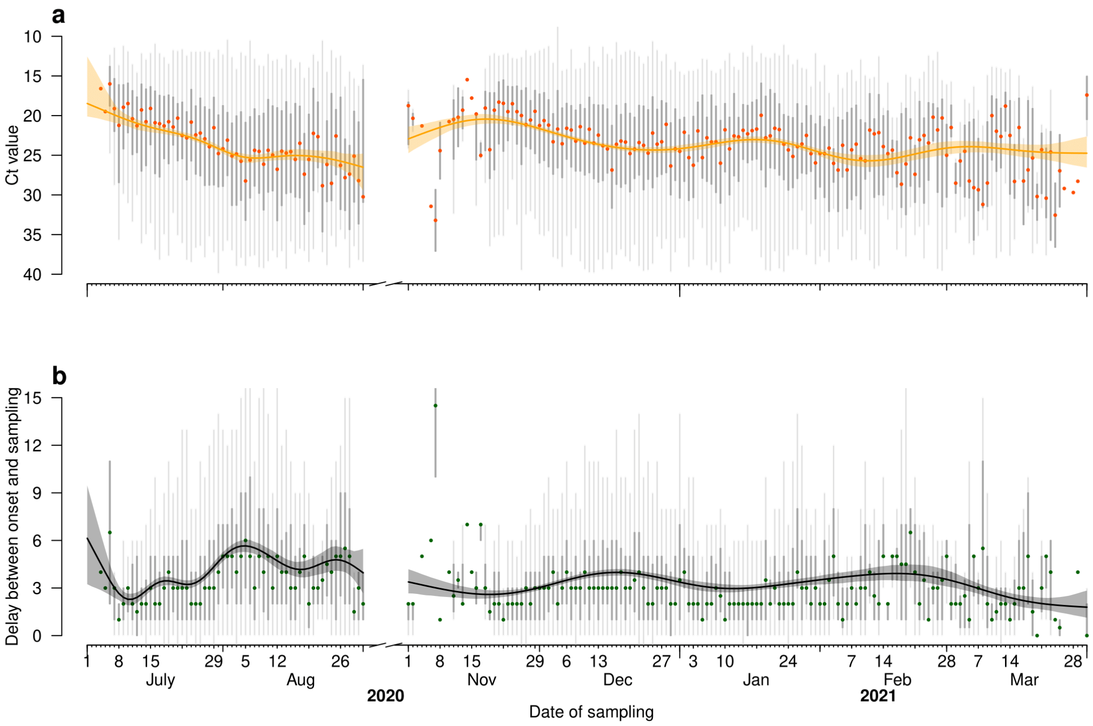

Supplementary Figure 1. Daily distributions of Ct values (a) and of delays from onset to sampling (b) by sampling date over the study period. a, Daily distributions of Ct values by sampling date. Orange lines and shaded areas represent the daily mean and 95% CIs of Ct values estimated from a GAM as in Eq. (2)(same as Fig. 1). **b,** Daily distributions of delays from onset to sampling by sampling date. Black lines and shaded areas represent the daily mean and 95% CIs of delays smoothed by GAM. Dark grey vertical lines and dots (red for daily Ct values in panel **a** and green for daily delays in panel **b**) show the interquartile range (IQR; defined as differences between 25th and 75th percentiles, same for other legends if not specified otherwise) and median of daily values, while light grey vertical lines represent the minimum and maximum of all values observed on that day (n = 9082 Ct records in panel **a** and n = 7217 onset-to-sampling delays in panel **b**).

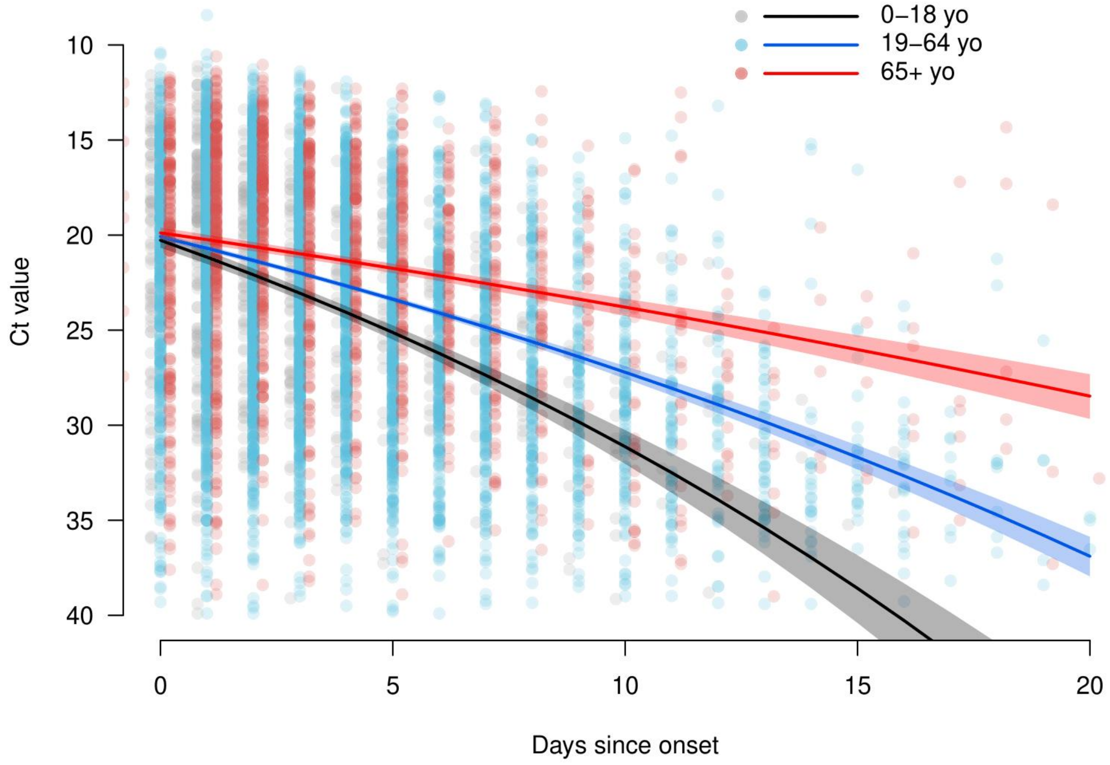

Supplementary Figure 2. Distribution of Ct values against delays between onset-to-sampling for different age groups. Dots show the first Ct value record for each individual against their time intervals between onset and sampling, lines and shaded areas represent mean and 95% CIs of the regression line for Ct values and the time-since-onset for corresponding age groups; colors indicated various age groups (0-18, 19-64 and 65+ years old).

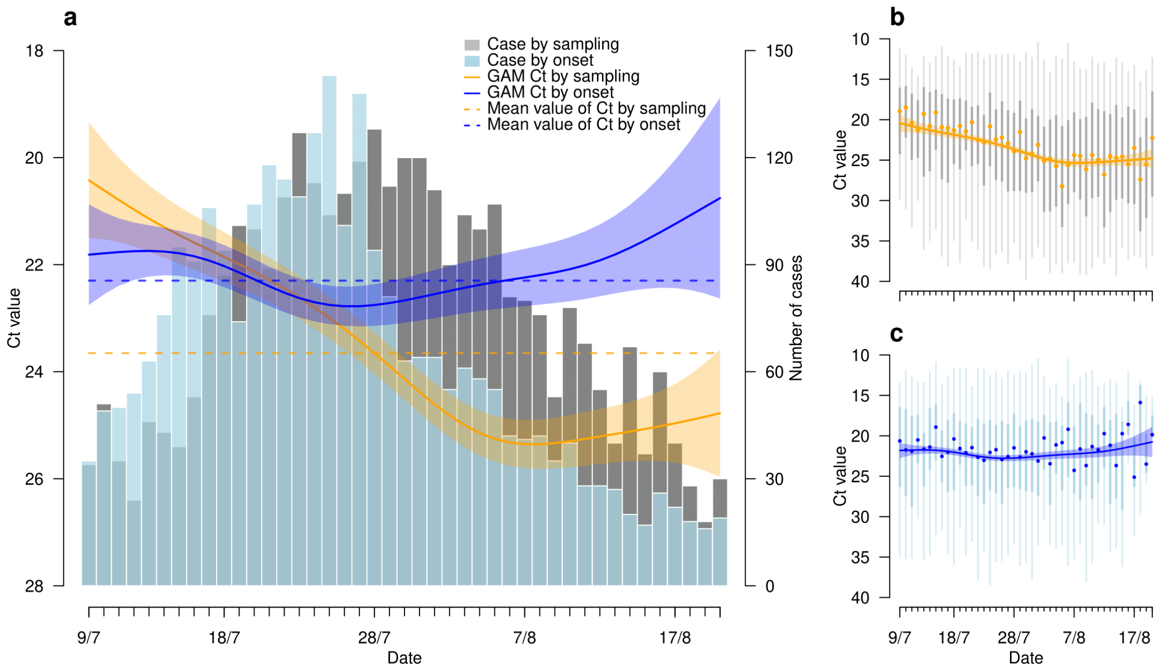

Supplementary Figure 3. Temporal distributions of Ct values at sampling and at onset during the third wave when sample sizes were sufficient (defined as >30 records per day). a, Temporal distribution of Ct values at sampling and at onset during the selected time period for comparison. Grey and azure bars indicate the number of cases by date of sampling and by date of onset respectively. Lines and corresponding shaded areas represent the mean and 95% CIs of daily Ct values at sampling (orange solid line) and at onset (blue solid line) that were estimated from a GAM over the compared period, as in Eq. (2) and (4) respectively (same for **b-c**), while the orange and blue dashed lines indicate the mean value of all Ct records by sampling and by onset over the compared time period. **b-c,** More detailed daily distribution of Ct values at sampling (**b**) and at onset (**c**). Boxes and colored dots show the IQR and median of Ct values per day, while light-colored vertical lines represent the minimum and maximum of all Ct values on that day ($n = 3263$ Ct records for panel **b** and $n = 2690$ Ct records for panel **c**).

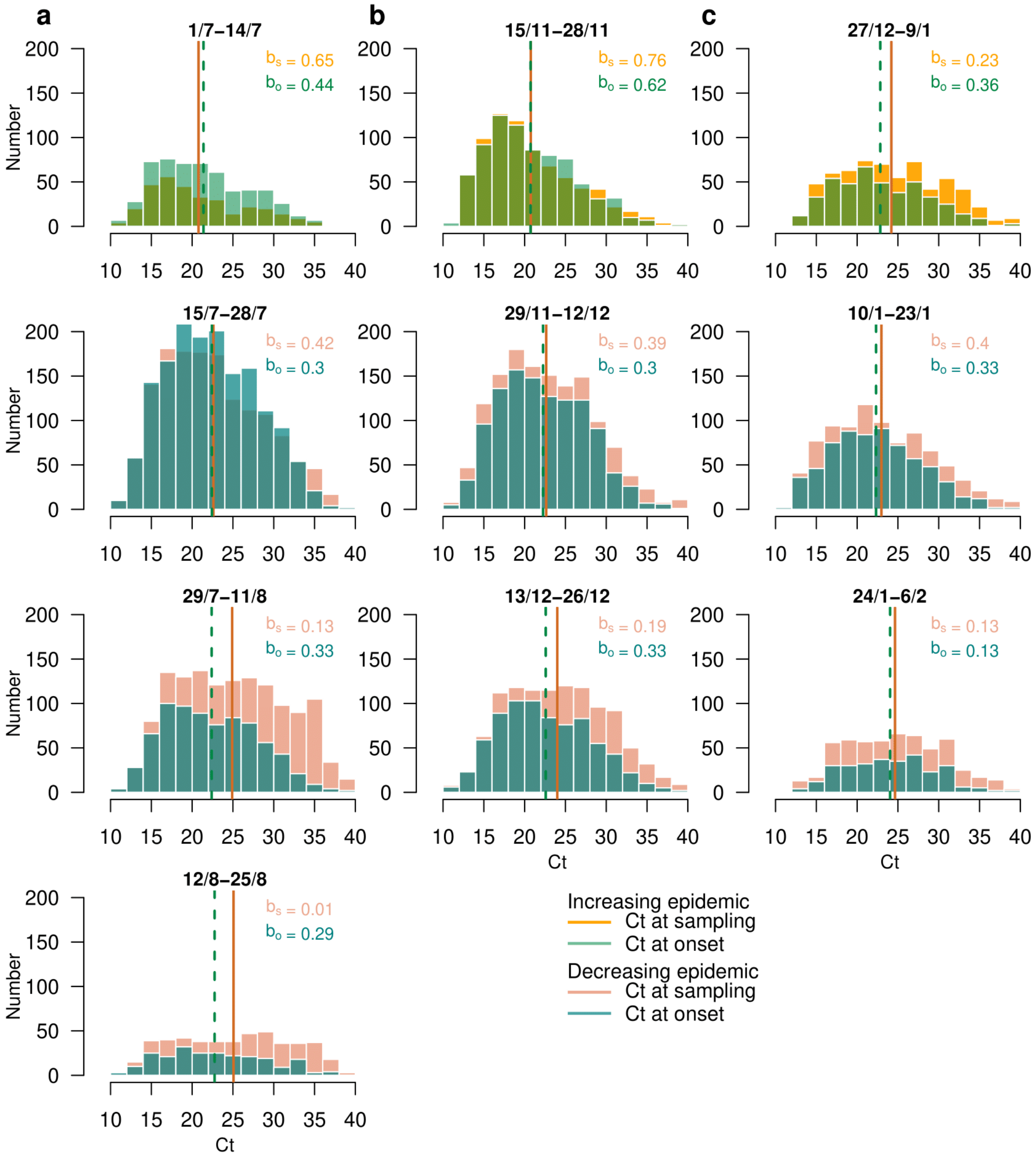

Supplementary Figure 4. Bi-weekly distribution of Ct values by date of sampling and of onset during the third (a) and fourth (b-c) wave of COVID-19 in Hong Kong. The Ct value shown by date of sampling was the actual value as sampled, whereas the Ct value shown by date of onset was the extrapolated value estimated from Eq. (4). The distribution of Ct values at sampling and at onset was illustrated in green and orange if during periods with increasing epidemic and in cyan and pink if during periods with decreasing epidemic. Solid and dashed vertical lines indicate mean values of all Ct values at sampling and at onset during the time window respectively. The skewness of Ct distribution at sampling (b_s) and at onset (b_o) over the biweekly period was shown at top-right of each panel with corresponding colors, while the exact time period was specified above each panel.

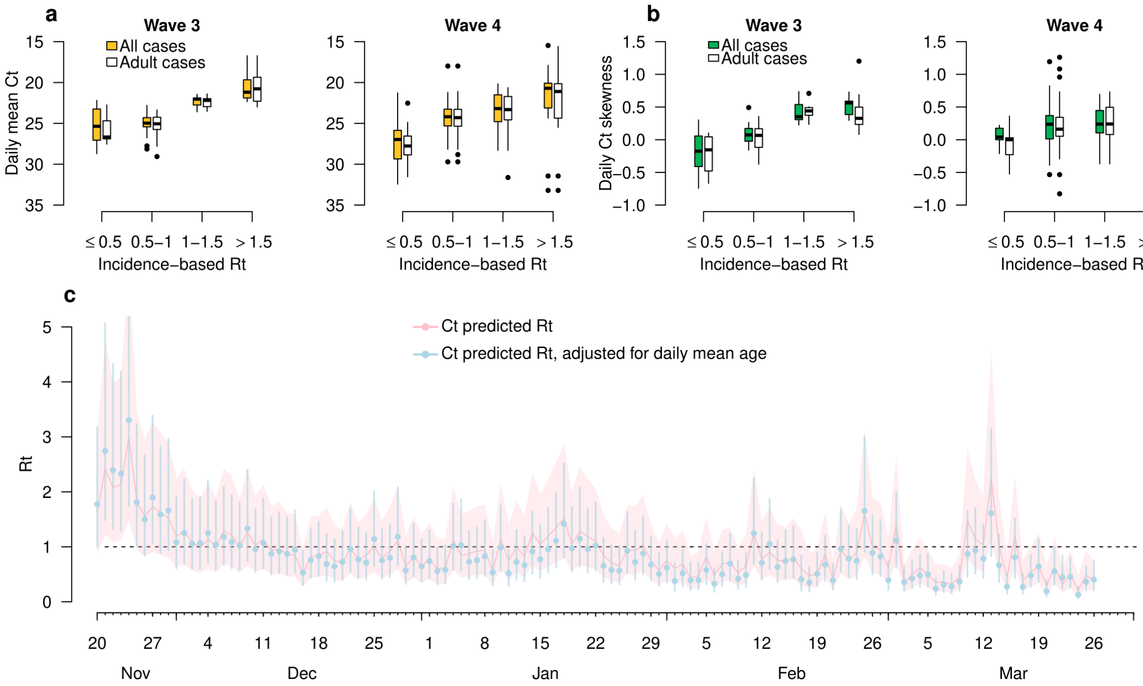

Supplementary Figure 5. Consistent Ct-based R_t estimates before and after adjusting for the age distribution of sampled cases. a-b, Comparison of daily mean Ct (**a**) and Ct skewness (**b**) under various incidence-based R_t intervals during July 2020 - August 2020 (wave 3) and November 2020 - March 2021 (wave 4). Boxes show the IQR and median of the corresponding Ct distribution derived from all samples (colored) and from samples of adult cases (white) respectively. Lower whiskers represent either the minimum or the smallest observed values that are within the distance of 1.5 times the IQR, upper whiskers represent either the maximum or largest observed values that are within the distance of 1.5 times the IQR of all daily Ct distributions under various incidence based R_t intervals, and dots represent values beyond the lower and upper whiskers (n = 59 and 146 daily Ct mean for wave 3 and 4 in panel **a**, and n = 57 and 138 daily Ct skewness for wave 3 and 4 in panel **b** respectively). **c**, Comparison of Ct-based R_t estimates before and after adjustment of age. Pink lines and shaded areas show the mean and 95% prediction intervals of Ct-based R_t estimated from the main model (Eq. (7)), while light blue dots and vertical lines show the mean and 95% prediction intervals of Ct-based R_t estimated after adjusting for the mean age of daily sampled cases (as in Eq. (8); n = 127 daily values). Black dotted line indicates the reference of R_t being 1.

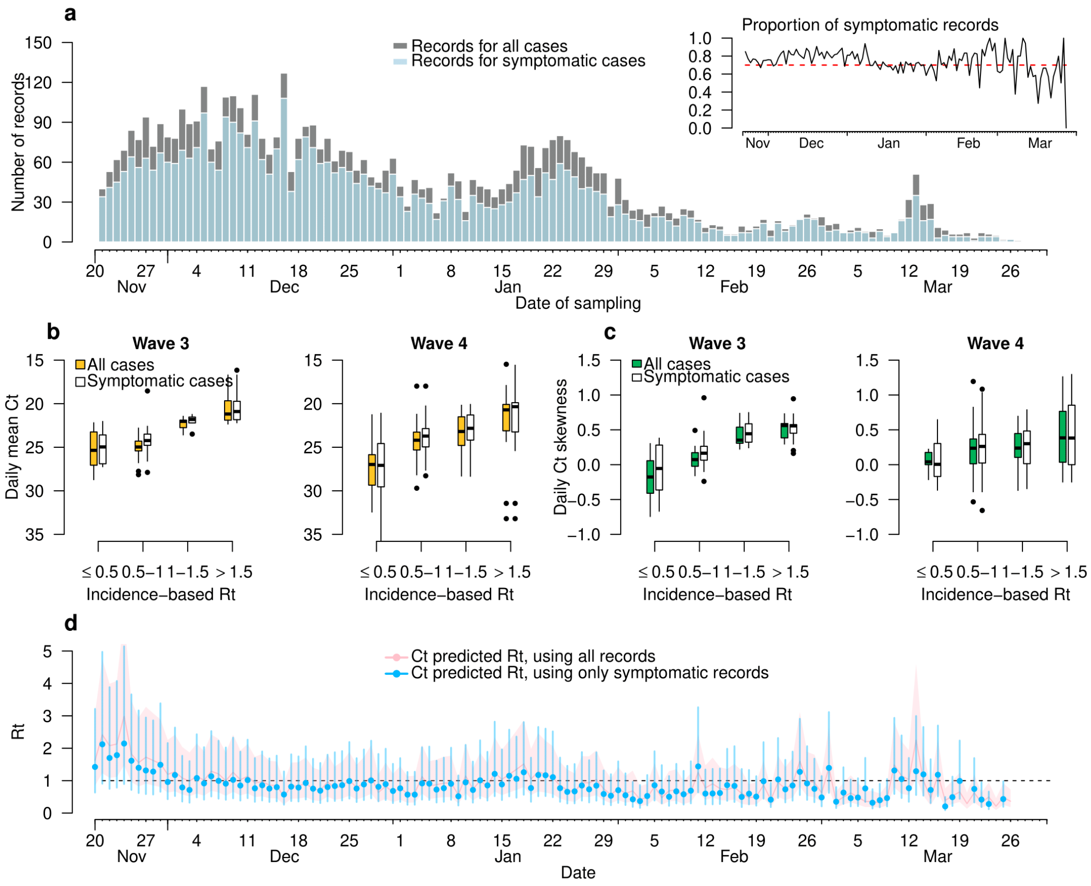

Supplementary Figure 6. Consistent Ct-based R_t estimates using either all records or using only records from symptomatic cases. a, Daily number of Ct values by date of sampling for all cases (grey bars) and for symptomatic cases (light blue bars) over wave 4. Top-right panel showed the proportion of symptomatic records over all records on each sampling date, with the red dotted line being the proportion of 0.7 as a reference. **b-c**, Comparison of daily mean Ct (**b**) and Ct skewness (**c**) under various incidence-based R_t intervals during July 2020 - August 2020 (wave 3) and November 2020 - March 2021 (wave 4). Boxes show the IQR and median of corresponding Ct distributions derived from all records (colored) and from records of symptomatic cases (white) respectively. Lower whiskers represent either the minimum or the smallest observed values that are within the distance of 1.5 times the IQR, upper whiskers represent either the maximum or largest observed values that are within the distance of 1.5 times the IQR of all daily Ct distributions under various incidence based R_t intervals, and dots represent values beyond the lower and upper whiskers (n = 59 and 146 daily Ct mean for wave 3 and 4 in panel **b**, and n = 57 and 138 daily Ct skewness for wave 3 and 4 in panel **c** respectively). **d**, Comparison of Ct-based R_t estimates using all records or using records from symptomatic cases, over the testing period (i.e., wave 4). Pink lines and shaded areas show the mean and 95% prediction intervals of Ct-based R_t estimated from the main model (Eq. (7)) using all records, while blue dots and vertical lines show the mean and 95% prediction intervals of Ct-based R_t estimated using symptomatic records only (also as in Eq. (7); n = 124 daily values). Black dotted line indicates the reference of R_t being 1.

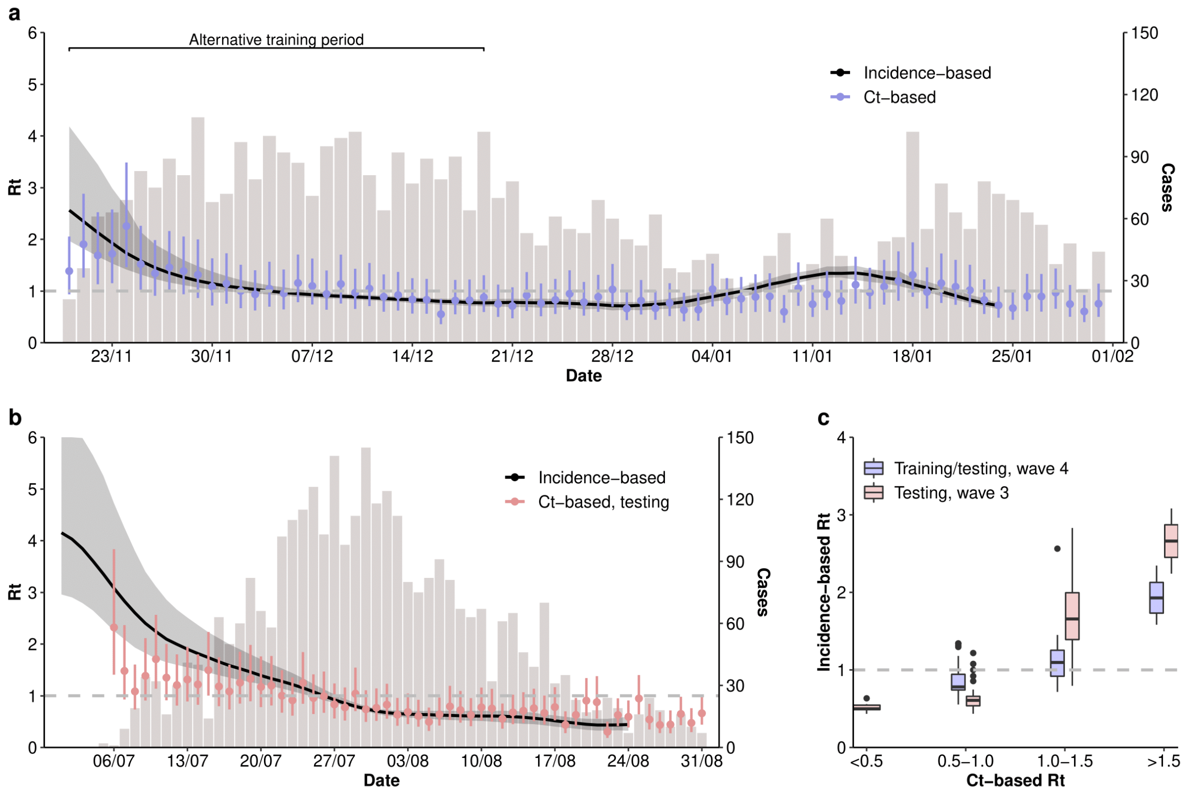

Supplementary Figure 7. Validation of the Ct-based model using an alternative training period in wave 4. Grey dotted lines indicate the reference of R_t being 1. **a-b,** Comparison of incidence-based and Ct-based R_t over the alternative training period (20 November – 19 December 2020) in wave 4 in predicting the latter half of the wave 4 (**a**) and in reversely predicting wave 3 (**b**). Black lines and shaded areas indicate the mean and 95% CrIs for incidence-based R_t, while colored dots and vertical lines represent the mean and 95% prediction intervals of Ct-based R_t ($n = 73$ and 57 daily values for the demonstrated days in wave 4 (in panel **a**) and for wave 3 (in panel **b**) respectively (same for panel **c**)). **c**, Consistency between the incidence-based and Ct-based R_t estimated using the alternative training period over wave 4 (purple) and wave 3 (pink). Boxes represent the IQR and median of incidence-based R_t under the corresponding Ct-based R_t intervals, lower whiskers represent the minimum and upper whiskers represent either the maximum or the largest observed values that are within the distance of 1.5 times the IQR of all incidence-based R_t under that Ct-based R_t interval, dots represent values beyond the lower and upper whiskers.

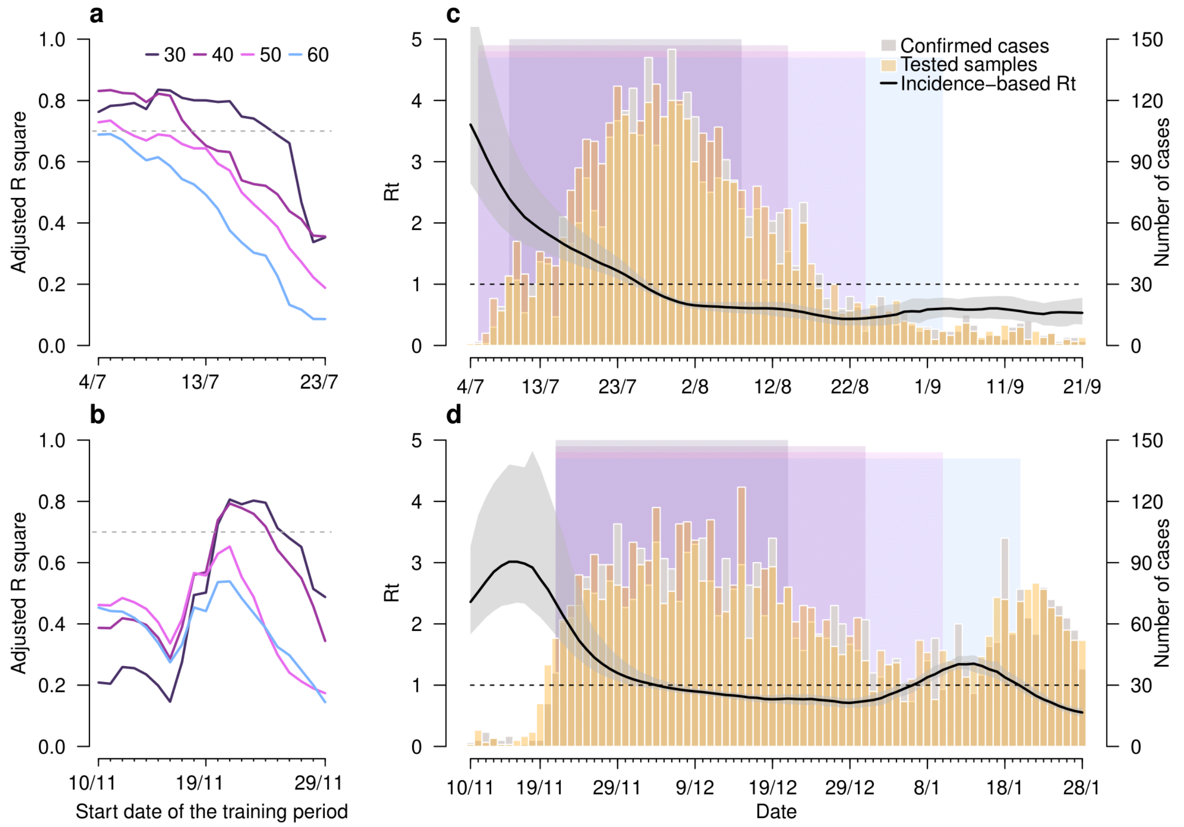

Supplementary Figure 8. Performance of models fitted over various length and timing of training periods. a-b, Performance of models fitted over different training periods with various durations and starting dates, over **a**) July-September 2020 and **b**) November 2020-January 2021. Color lines show the performance of models (measured by adjusted R square) fitted over training periods with a duration of 30-60 days respectively, with the starting date of the training period specified in the x-axes. The grey dashed line in the background referred to adjusted R square being 0.7, as a reference for comparison. **c-d,** Time period covered by the model with the largest adjusted R square over **c**) July-September 2020 and **d**) November 2020-January 2021. Grey and orange bars represent the number of laboratory-confirmed cases (by date of reporting) and of sampled collections (by date of sampling) respectively. Black lines and shaded areas indicate the mean and 95% CrIs for incidence-based R_t. Time periods used for fitting the model with the largest adjusted R square among all models fitted over that certain length of training period was indicated by corresponding colored backgrounds (same color code as in **a**). Black dotted lines indicate the reference of R_t being 1.

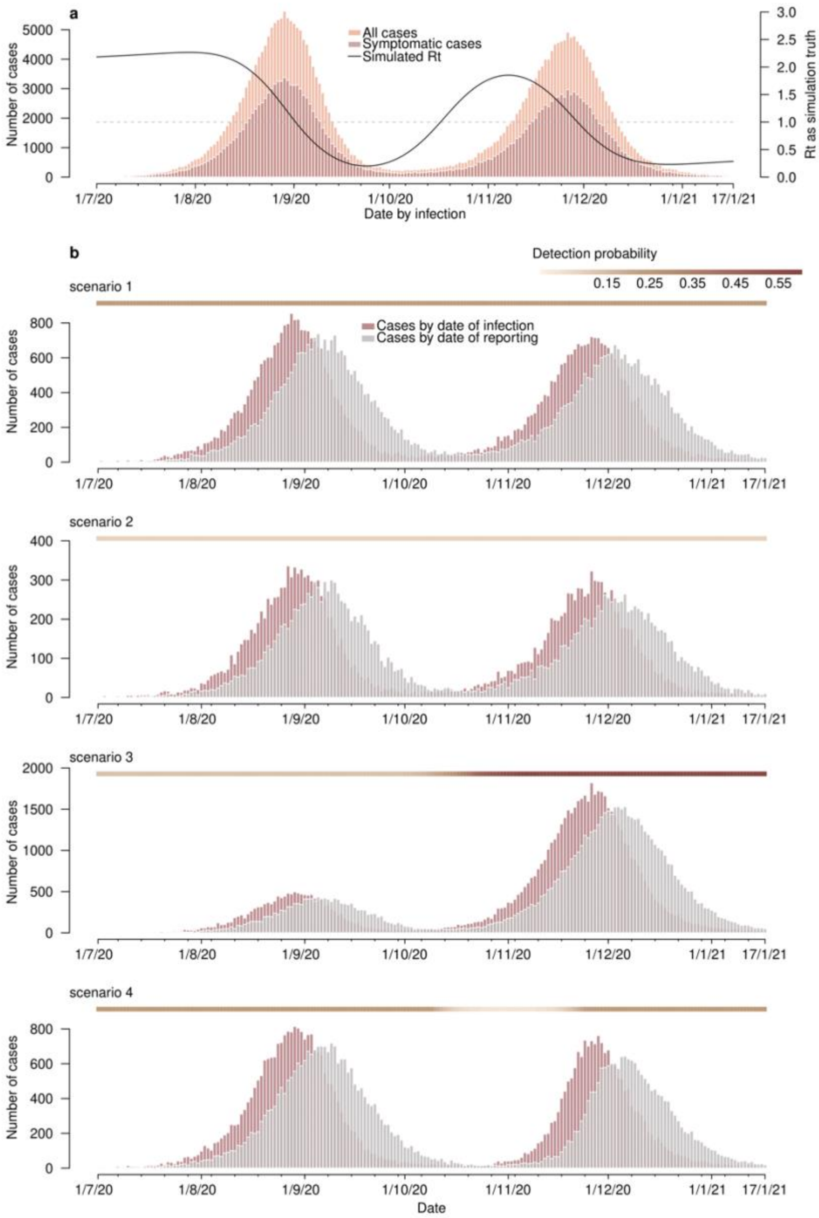

Supplementary Figure 9. Simulated incidence curve (a) and epidemic curves under various scenarios of case detection (b). a, Daily number of cases by date of infection and R_t as simulated using the SEIR model (i.e., simulation truth). Orange and dark red bars represented the number of all infected cases and of symptomatic cases by date of infection, while the black line indicates the R_t as simulation truth (as in Eq. (12)). Grey dotted line indicates the reference of R_t being 1. **b**, Number of cases that would have been detected under each scenario. Dark red and grey bars represented number of cases by date of infection and by date of reporting respectively. Daily detection probability was indicated in the colormap above each panel, with deeper color indicating higher detection proportion and lighter color the reverse (as shown in the legend on the top right for panel **b**). We synthesized the practice of stable detection (detection probability fixed at 25%; scenario 1), limited but stable detection (detection probability fixed at 10%; scenario 2), varying detection (detection probability increased from 15% to 60% in the second wave; scenario 3) and under detection (detection probability dropped to 5% for certain days, with the detection probability fixed at 25% for other days; scenario 4)(same for **Supplementary Figure 10**).

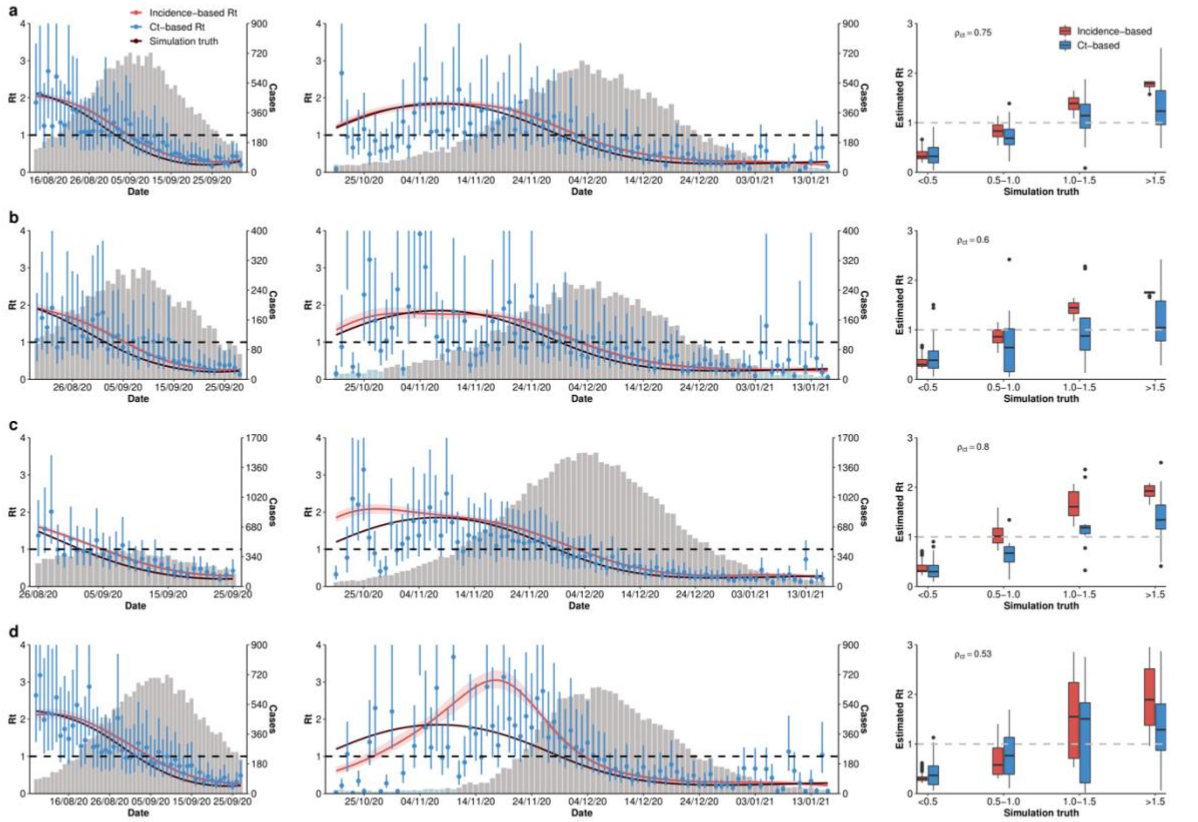

Supplementary Figure 10. Consistency between incidence-based and Ct-based R_t under each synthesized scenario over a representative simulation run. a-d represent situations in scenarios 1-4 respectively, with left and middle columns showing simulation truth, incidence-based and Ct-based R_t estimates in the first wave (training period; left columns) and second wave (middle columns) respectively and right columns showing distributions of incidence-based (red boxes) and Ct-based (blue boxes) R_t estimates under various intervals of simulation truth over the testing period in each scenario. In panels shown in left and middle columns, black line show the R_t taken as the simulation truth (estimated from Eq. (12)), red lines and shaded areas represent the mean and 95% CIs of the incidence-based R_t as estimated by EpiNow2[3], while blue dots and vertical lines represent the mean and 95% prediction intervals of Ct-based R_t ($n = 51$, 41, 31 and 51 daily values for left panels from **a** to **d**, $n = 91$ daily values for all middle panels, while $n = 107$, 111, 114 and 112 for right panels from **a** to **d** respectively). Grey and light blue bars in left and middle columns indicate daily number of cases by date of reporting and whether the number was over 30 (grey bars) or not (light blue bars). In panels shown in the right column, boxes represent the IQR and median of incidence-based R_t under the corresponding Ct-based R_t intervals, lower whiskers represent the minimum and upper whiskers represent either the maximum or the largest observed values that are within the distance of 1.5 times the IQR of all incidence- or Ct-based R_t under that simulation truth interval, dots represent values beyond the lower and upper whiskers. The Spearman correlation coefficient between Ct-based R_t and the simulation truth over testing periods were indicated as ρ_{ct} in panels shown in right columns. Black dotted lines in left and middle columns and grey dotted line in right columns indicated the reference of R_t being 1.
