# Supplementary material for: Incorporating temporal distribution of population-level viral load enables real-time estimation of COVID-19 transmission
Source: Nat Commun. 2022 Mar 3;13:1155. doi: 10.1038/s41467-022-28812-9 (PMC8894407; doi:10.1038/s41467-022-28812-9)
Supplement: Supplementary file 3 — Reporting Summary [file 41467_2022_28812_MOESM3_ESM.pdf]

nature portfolio

Corresponding author(s): Benjamin J. Cowling

Last updated by author(s): Jan 30, 2022

Reporting Summary

Nature Portfolio wishes to improve the reproducibility of the work that we publish. This form provides structure for consistency and transparency in reporting. For further information on Nature Portfolio policies, see our Editorial Policies and the Editorial Policy Checklist.

Statistics

For all statistical analyses, confirm that the following items are present in the figure legend, table legend, main text, or Methods section.

n/a	Confirmed	
☐	☒	The exact sample size (*n*) for each experimental group/condition, given as a discrete number and unit of measurement
☐	☒	A statement on whether measurements were taken from distinct samples or whether the same sample was measured repeatedly
☐	☒	The statistical test(s) used AND whether they are one- or two-sided<br>*Only common tests should be described solely by name; describe more complex techniques in the Methods section.*
☐	☒	A description of all covariates tested
☐	☒	A description of any assumptions or corrections, such as tests of normality and adjustment for multiple comparisons
☐	☒	A full description of the statistical parameters including central tendency (e.g. means) or other basic estimates (e.g. regression coefficient) AND variation (e.g. standard deviation) or associated estimates of uncertainty (e.g. confidence intervals)
☐	☒	For null hypothesis testing, the test statistic (e.g. *F*, *t*, *r*) with confidence intervals, effect sizes, degrees of freedom and *P* value noted<br>*Give P values as exact values whenever suitable.*
☐	☒	For Bayesian analysis, information on the choice of priors and Markov chain Monte Carlo settings
☒	☐	For hierarchical and complex designs, identification of the appropriate level for tests and full reporting of outcomes
☐	☒	Estimates of effect sizes (e.g. Cohen's *d*, Pearson's *r*), indicating how they were calculated

Our web collection on statistics for biologists contains articles on many of the points above.

Software and code

Policy information about availability of computer code

Data collection	The aggregate data of daily Ct mean and skewness among confirmed COVID-19 cases were derived from hospitalization records of confirmed COVID-19 cases from the e-record system managed by the Hospital Authority in Hong Kong, with the daily Ct skewness calculated using the R package "e1071" (version 1.7-9).
Data analysis	All statistical analyses were conducted in R version 4.1.2 (R Development Core Team, 2021). Incidence-based Rt from synthetic case count data was estimated using the R package "EpiNow2" (version 1.3.2). All R codes and full lists of packages used have been provided in the Github repository (https://github.com/vanialin/ct_rt_hk).

For manuscripts utilizing custom algorithms or software that are central to the research but not yet described in published literature, software must be made available to editors and reviewers. We strongly encourage code deposition in a community repository (e.g. GitHub). See the Nature Portfolio guidelines for submitting code & software for further information.

Data

Policy information about availability of data

All manuscripts must include a data availability statement. This statement should provide the following information, where applicable:

- Accession codes, unique identifiers, or web links for publicly available datasets
- A description of any restrictions on data availability
- For clinical datasets or third party data, please ensure that the statement adheres to our policy

We obtained demographic and epidemiological information of confirmed COVID-19 cases from the Centre for Health Protection, Department of Health of the Government of Hong Kong (https://www.coronavirus.gov.hk/eng/index.html), including age, date of symptom onset and case classification (i.e., local, imported and

contacts of local or imported cases). Daily aggregate data (including case counts, incidence-based Rt and Ct distributions) and simulation data generated in this study have been deposited in the GitHub repository (https://github.com/vanialin/ct_rt_hk/tree/main/data).

Field-specific reporting

Please select the one below that is the best fit for your research. If you are not sure, read the appropriate sections before making your selection.

☒ Life sciences ☐ Behavioural & social sciences ☐ Ecological, evolutionary & environmental sciences

For a reference copy of the document with all sections, see nature.com/documents/nr-reporting-summary-flat.pdf

Life sciences study design

All studies must disclose on these points even when the disclosure is negative.

Sample size	The study was based on daily numbers of confirmed COVID-19 cases in Hong Kong as well as their relevant epidemiological information and hospitalization records, which generated the effective reproductive number and population Ct distributions. No sample size calculation was needed.
Data exclusions	We excluded information on imported cases as well as Ct records over 40 (which was regarded as cut-off for a negative Ct value) as we mainly focused on the evaluation of local transmissions using Ct values from positive RT-qPCR test results.
Replication	We confirmed replications for all analyses can be performed with data and codes provided in https://github.com/vanialin/ct_rt_hk.
Randomization	This is an observational study and no randomization is needed.
Blinding	This is an observational study and no blinding is performed.

Reporting for specific materials, systems and methods

We require information from authors about some types of materials, experimental systems and methods used in many studies. Here, indicate whether each material, system or method listed is relevant to your study. If you are not sure if a list item applies to your research, read the appropriate section before selecting a response.

Materials & experimental systems

n/a	Involved in the study	
☒	☐	Antibodies
☒	☐	Eukaryotic cell lines
☒	☐	Palaeontology and archaeology
☒	☐	Animals and other organisms
☒	☐	Human research participants
☒	☐	Clinical data
☒	☐	Dual use research of concern

Methods

n/a	Involved in the study	
☒	☐	ChIP-seq
☒	☐	Flow cytometry
☒	☐	MRI-based neuroimaging
